# Supplementary material for: Factors Associated with Mortality in Nosocomial Lower Respiratory Tract Infections: An ENIRRI Analysis
Source: Antibiotics (Basel). 2025 Jan 26;14(2):127. doi: 10.3390/antibiotics14020127 (PMC11851455; doi:10.3390/antibiotics14020127)
Supplement: Supplementary file 1 [file antibiotics-14-00127-s001.zip › antibiotics-3366166-supplementary.pdf]

Supplement Material

SUPPLEMENT FIGURES

e-Figure S1. Directed Acyclic Graph (DAG) used for the logistic regressions.

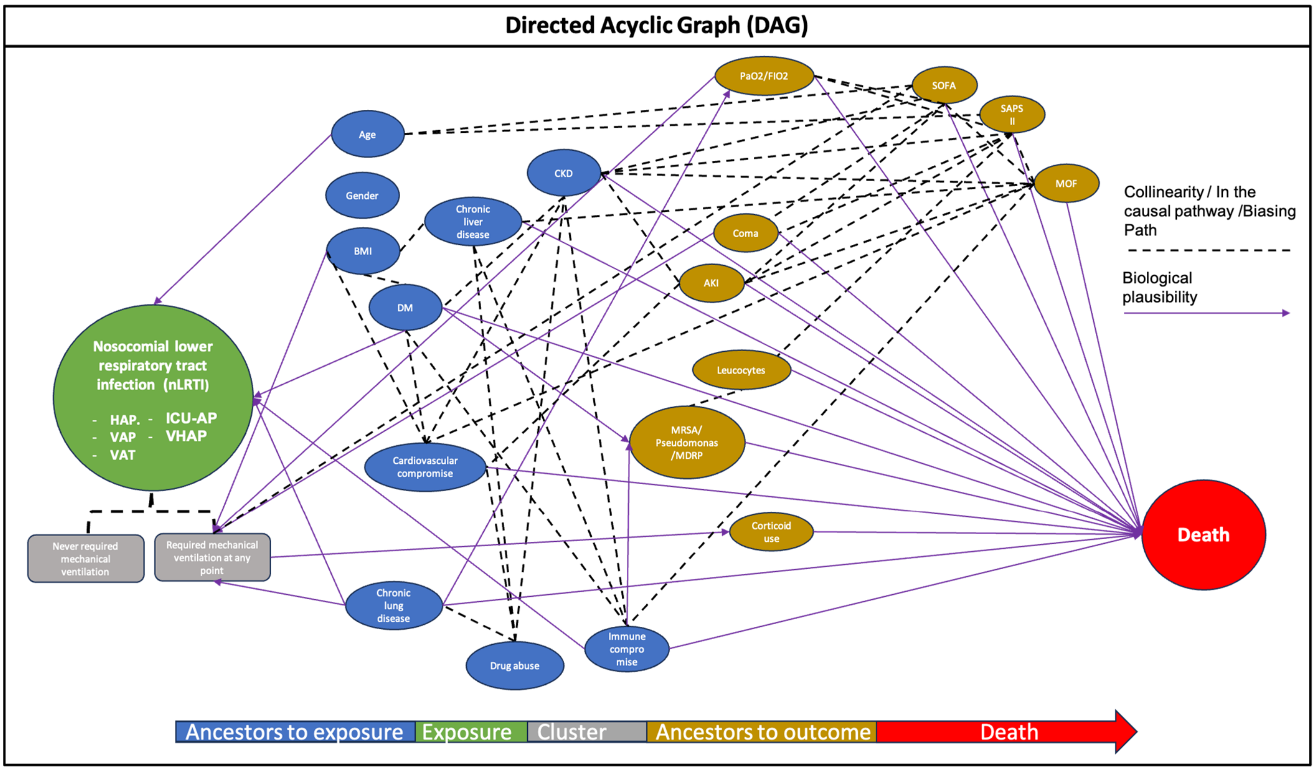

e-Figure S2. A) Random Forrest classification model including demographic and baseline characteristics and their feature importance **B)** ROC Curve and AUC for Random Forest Clustering Model: ICU vs. Non-ICU Acquired Pneumonia

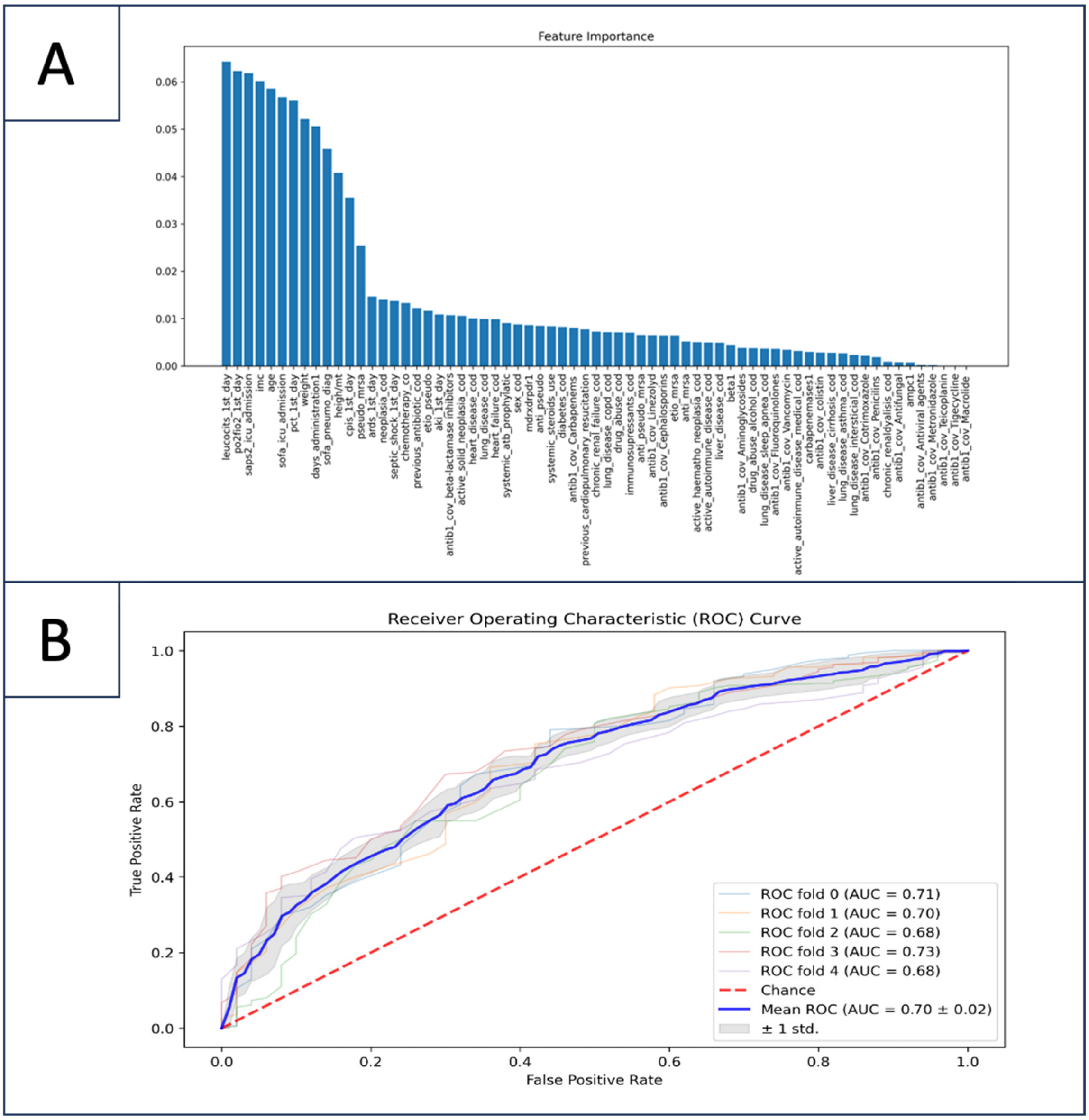

e-Figure S3. ROC Curve and AUC for Random Forest Clustering Model: ICU vs. Non-ICU Acquired Pneumonia.

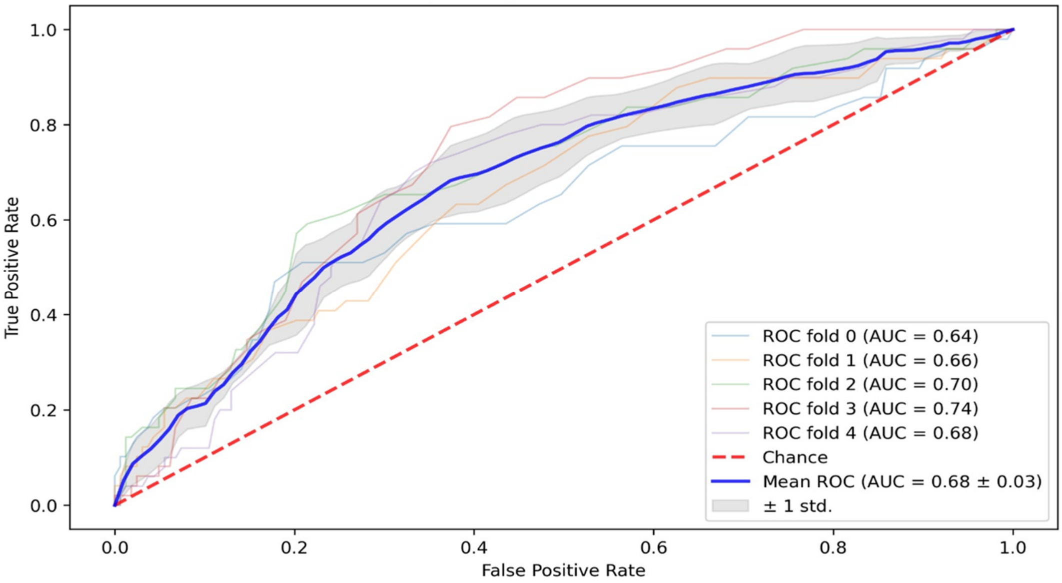

SUPPLEMENT TABLES

e-Table S1. LRTI specific subgroup classification among non-ventilated and ventilated patients.

LRTI sub-group	Invasive mechanical ventilation	
	No<br>N=250	Yes<br>n=810
VAP	0 (0.0)	556 (68.6)
VAT	0 (0.0)	160 (19.8)
HAP	152 (60.8)	0 (0.0)
VHAP	0 (0.0)	94 (11.6)
ICU HAP	98 (39.2)	0 (0.0)

Data are presented as No. (%)
VAP: Ventilator-Associated Pneumonia; VAT: Ventilator Associated Tracheitis, HAP: Hospital-Acquired Pneumonia, VHAP: HAP that required invasive ventilation, ICU HAP: Intensive Care Unit HAP.

e-Table S2. Bivariate and multivariate analysis for 28 days mortality in the whole cohort.

Variable	Bivariate		Multivariate	
	OR (95% IC)	p-value	OR (95%IC)	p-value
Demographic				
Age	1.02 (1.01-1.04)	**<0.001**	1.02 (1.00-1.03)	**0.01**
Gender (male)	1.16 (0.82-1.65)	0.43		
BMI	1.02 (0.99-1.04)	0.17		
Comorbidities				
Diabetes Mellitus	1.28 (0.89-1.84)	0.21	0.95 (0.64-1.41)	0.79
Chronic Kidney Disease	1.48 (0.95-2.31)	0.09	0.99 (0.59-1.65)	0.96
Immunocompromised Disease	0.86 (0.60-1.23)	0.47		
Chronic Heart Disease	1.28 (0.92-1.79)	0.16	1.05 (0.71-1.54)	0.81
Chronic Liver Disease	1.64 (0.93-2.88)	0.10	1.74 (0.95-3.19)	0.07
Lung Disease	1.23 (0.87-1.76)	0.26		
Drug Abuse	0.79 (0.53-1.18)	0.28		
Severity				
SAPS II (Pneumonia Diagnosis)	1.044 (1.04-1.05)	**<0.001**	1.04 (1.03-1.05)	**<0.001**
Systemic Corticoid Use	1.04 (1.00-2.05)	0.86		
Coma	0.97 (0.68-1.39)	0.93		
IMV	1.54 (1.04-2.28)	**0.03**	1.53 (1.01-2.32)	**0.04**
Complications				
Acute Kidney Injury	1.43 (1.00-2.05)	**0.05**		
Microorganism and Antibiotic-Resistance Pattern				
Pseudomonas	0.73 (0.45-1.21)	0.28		
MRSA	1.50 (0.78-2.89)	0.27		
MDRP	0.87 (0.60-1.25)	0.47		
SOFA: Sequential Organ Failure Assessment, MDR: Multidrug-Resistant Pathogens, PDR: Pan Drug-Resistant Pathogens, ICU: Intensive Care Units.				

e-Table S3. Bivariate and multivariate analysis for 90 days mortality in the whole cohort.

Variable	Bivariate		Multivariate	
	OR (95% IC)	p-value	OR (95%IC)	p-value
Demographic				

Age	1.03 (1.02-1.04)	**<0.001**	1.02 (1.01-1.03)	**<0.001**
Gender (male)	0.94 (0.71-1.26)	0.72		
BMI	1.01 (0.99-1.03)	0.51		
Comorbidities				
Diabetes Mellitus	1.47 (1.08-1.99)	**0.02**	1.05 (0.75-1.47)	0.78
Chronic Kidney Disease	2.06 (1.40-3.02)	**<0.001**	1.43 (0.92-2.21)	0.11
Immunocompromised Disease	1.23 (0.92-1.65)	0.15		
Chronic Heart Disease	1.55 (1.17-2.05)	**<0.001**	1.15 (0.83-1.60)	0.39
Chronic Liver Disease	1.57 (0.95-2.61)	0.08	1.72 (1.00-2.95)	**0.05**
Lung Disease	1.13 (0.83-1.52)	0.44		
Drug Abuse	0.86 (0.62-1.19)	0.41		
Severity				
SAPS II (Pneumonia Diagnosis)	1.04 (1.03-1.05)	**<0.001**	1.03 (1.02-1.04)	**<0.001**
Systemic Corticoid Use	1.45 (1.08-1.94)	**0.02**		
Coma	1.25 (0.93-1.68)	0.15		
IMV	1.22 (0.90-1.66)	0.22	1.26 (0.91-1.75)	<0.15
Complications				
Acute kidney injury	1.73 (1.28-2.35)	**0.001**		
Microorganism and Antibiotic-Resistance Pattern				
Pseudomonas	0.87 (0.59-1.27)	0.50		
MRSA	1.14 (0.64-2.02)	0.66		
MDRP	1.28 (0.96-1.71)	0.10		
SOFA: Sequential Organ Failure Assessment, MDR: Multidrug-Resistant Pathogens, PDR: Pan Drug-Resistant Pathogens, ICU: Intensive Care Units.				
